# Single-Parent Family Structure and Children’s BMI: The Mediating Role of Socioeconomic Status

**DOI:** 10.3390/children13101336

**Published:** 2026-10-01

**Authors:** Gorana Jerković, Sanja Musić Milanović, Ivana Unić Šabašov

**Affiliations:** 1 Department of Paediatrics, University Hospital Centre Split, School of Medicine, University of Split, Spinčićeva 1, 21000 Split, Croatia; 2 Division for Health Promotion, Croatian Institute of Public Health, Rockenfellerova 7, School of Medicine, University of Zagreb, 10000 Zagreb, Croatia

**Keywords:** childhood obesity, family structure, mediation analysis, single-parent families, socioeconomic status

## Abstract

**Highlights:**

**What are the main findings?**

- In a national sample of 5425 Croatian children aged 6–10, those from single-parent families had lower socioeconomic status (SES), and lower SES was linked to higher BMI z-scores.
- SES fully mediated the link between single-parent family structure and childhood BMI, as the indirect effect through SES was significant, while the direct effect of family structure alone was not.

**What are the implications of the main findings?**

- Elevated obesity risk in children from single-parent households appears to stem largely from economic disadvantage rather than family structure itself.
- Obesity prevention efforts should move beyond individual/behavioral approaches and incorporate policies that reduce socioeconomic inequality among single-parent families.

**Abstract:**

Objectives: Previous research has consistently shown that children living in single-parent families are at greater risk of overweight and obesity. However, the mechanisms underlying this association remain insufficiently understood. Socioeconomic disadvantage may represent an important pathway linking family structure to children’s weight outcomes. This study examined whether socioeconomic status (SES) mediates the association between single-parent family structure and children’s BMI z-scores. Methods: Data were obtained from the Croatian Childhood Obesity Surveillance Initiative (CroCOSI) conducted during the 2021/2022 school year. The sample comprised 5425 children aged 6–10 years. Family structure was specified as the independent variable, BMI z-score as the outcome variable, and SES as the mediator. Age, gender, region, and urbanicity were included as covariates in the mediation model. Results: Children living in single-parent families had significantly lower SES than children living in two-parent families. Higher SES was associated with lower BMI z-scores. The indirect effect of family structure on BMI z-score through SES was statistically significant, whereas the direct effect was not statistically significant. These findings indicate that SES mediates the association between single-parent family structure and children’s BMI. Conclusions: The findings suggest that socioeconomic disadvantage is an important mechanism linking single-parent family structure to childhood weight outcomes. Efforts to reduce childhood obesity should therefore extend beyond individual behavioral interventions and address broader socioeconomic inequalities affecting families. Policies aimed at improving the socioeconomic conditions of single-parent families may contribute to reducing disparities in childhood obesity and promoting healthier developmental outcomes among children.

## 1. Introduction

Childhood obesity represents one of the most pressing public health crises of the 21st century. Pediatric obesity constitutes a significant health burden throughout the developmental period and demonstrates strong associations with deleterious health and psychosocial outcomes extending into adulthood, according to the World Health Organization (2020) [1].

Pediatric obesity predisposes individuals to a constellation of metabolic and cardiovascular complications, including insulin resistance and type 2 diabetes mellitus, hypertension, and dyslipidemia [2,3,4]. Its consequences also extend into adulthood: adolescent obesity has been associated with a substantially higher risk of chronic kidney disease in young adulthood [5] and with infertility and childlessness among women [6].

The previously established WHO 2025 obesity target—maintaining stable prevalence rates between 2010 and 2025—has been surpassed by most nations globally [7,8,9,10,11,12]. The most recent estimates from the NCD Risk Factor Collaboration (NCD-RisC; NCD, noncommunicable disease), based on measured height and weight data, indicate that, in 2022, 9.3% of boys and 6.9% of girls aged 5–19 years were living with obesity, corresponding to approximately 159 million school-aged children and adolescents worldwide [13]. Using different methods and age groups, the Global Burden of Disease (GBD) 2021 study estimated that 93.1 million children aged 5–14 years and 80.6 million adolescents and young adults aged 15–24 years had obesity in 2021. It also found that obesity prevalence in these age groups had tripled since 1990 and forecast further increases to 2050 [12]. A recent meta-analysis of 2033 studies from 154 countries similarly reported a 1.5-fold increase in childhood obesity prevalence between 2000–2011 and 2012–2023 [14].

The etiology of pediatric overweight and obesity encompasses both modifiable and non-modifiable risk determinants. Modifiable factors include inadequate physical activity, sedentary behaviors, socioeconomic disadvantage, suboptimal dietary patterns, and adverse environmental exposures [2,3]. The predominant non-modifiable determinant is genetic predisposition, with substantially elevated obesity risk observed among offspring of parents with overweight or obesity [15,16,17,18].

Interventions for reducing childhood obesity aimed at modifiable risk factors have been extensively indicated in the literature as key to reversing the detrimental health effects that impact the quality of life in children [19,20]. Interventions for children and young adolescents typically need to be targeted at school, at home, and in the community, whereas older adolescents typically need to be targeted outside of school [19,20,21,22].

The evolving landscape of family structures in contemporary society has significant implications for childhood obesity prevalence. Demographic trends demonstrate a substantial increase in single-parent households, with single-mother families having increased from 3 million in 1970 to 10 million in 2003 (12% to 36%), while single-father families increased from less than half a million to 2 million (1% to 6%) within the same period [23,24,25]. This structural transformation reflects broader societal changes, including delayed marriage, increased non-marital births, and elevated divorce rates, all contributing to family instability during critical developmental periods.

The transition to single-parent status introduces multifaceted challenges that directly impact children’s weight trajectories [26]. Parental income is theorized to be negatively associated with childhood obesity, specifically by enabling parents to purchase healthy, nutritious foods and to engage their children in extra-curricular physical activities [26]. Simultaneously, the increased time demands on single parents reduce capacity for food preparation, dietary monitoring, and establishment of structured health routines [27]. These resource limitations often necessitate reliance on convenient, processed foods and sedentary entertainment options, creating an obesogenic environment.

Beyond material constraints, family disruption generates psychological stress that influences eating behaviors and weight regulation [28]. Children experiencing parental separation or ongoing family conflict frequently exhibit emotional eating patterns, utilizing high-calorie comfort foods as coping mechanisms [29]. The quality of parent–child relationships and parenting practices during these transitions critically determine whether children develop adaptive or maladaptive responses to family stress, with implications for long-term weight status and metabolic health [23,24,27,28]. Single-parent households face distinctive psychosocial challenges that extend beyond economic constraints, creating complex pathways to childhood obesity. Role multiplicity in single-parent families generates chronic stress, as parents navigate competing demands of employment and childcare responsibilities without adequate support systems. This role strain manifests in compromised family routines, including irregular meal patterns, reduced family dining frequency, and increased screen time during meals—factors consistently associated with poor dietary quality and weight gain. Reciprocal reinforcing relationships among family members are important for acquiring and maintaining new behaviors [30]. The family is an ideal mutually reinforcing environment in which healthy behaviors can be introduced, accepted and maintained [31].

The intersection of single parenthood and socioeconomic disadvantage creates particularly vulnerable populations for childhood obesity. This economic vulnerability translates into dietary patterns characterized by higher consumption of energy-dense, nutrient-poor foods, increased intake of sugar-sweetened beverages, and elevated sedentary behaviors, including excessive screen time [32].

The inverse socioeconomic gradient in childhood obesity was first documented in 1972 by Stunkard et al., who demonstrated a sixfold elevation in obesity prevalence among American girls from low-income compared to affluent neighborhoods, with boys exhibiting a similar, though less pronounced, pattern [33]. This landmark finding established the foundation for extensive subsequent research across high-income nations, consistently documenting parallel phenomena across diverse populations and geographic contexts.

The relationship between SES and childhood obesity demonstrates complex racial and ethnic disparities that persist across income levels [34]. While obesity prevalence generally decreases with increasing household income among white children, this protective effect of higher SES is attenuated among racial and ethnic minority populations. For example, Goto et. al. (2022.) reported a widening socioeconomic disparity in adolescence obesity prevalence in the United States, with the difference in prevalence increasing by 6.4% from 1999 to 2018 by household income stratifications [35]. Moreover, childhood obesity prevalence in the United States is higher among racial/ethnic minority children [34]. In a study comparing 2011 to 2012 with 2017 to 2020, childhood obesity prevalence increased for Mexican American and non-Hispanic Black children, but not for non-Hispanic white children [36].

In the United Kingdom, children exposed to persistent poverty from infancy, particularly when combined with poor parental mental health, had a higher risk of obesity at age 14 years than children exposed to low levels of poverty and family adversity (aOR 1.8) [37]. Similarly, in a Canadian birth cohort, children from persistently poor households were 2.2 to 3.0 times more likely to be overweight or obese at ages 8, 10, and 12 years than children from persistently non-poor households, with the association strengthening with age [38]. In terms of a broader definition of poverty, material deprivation (defined as both poverty and parental unemployment) was associated with slightly increased BMI among children in Denmark [39].

These disparities suggest that socioeconomic factors alone cannot fully explain obesity patterns, highlighting the need to examine additional mechanisms including neighborhood environments, cultural factors, food accessibility, and systemic barriers to health-promoting resources.

The proposed mediation model is grounded in the Family Investment Model and the Family Stress Model [40]. According to the Family Investment Model, economic resources shape the material and time investments parents can make in their children’s health, such as nutritious food, organized sport, and safe spaces for play [41]. According to the Family Stress Model, economic hardship increases parental psychological distress, which undermines parenting practices and household routines related to eating and activity. Single-parent families typically have one income and one adult available for time investments, so family structure is expected to affect children’s weight largely through its impact on the family’s socioeconomic position. From this perspective, SES is not merely a confounder to be controlled for, but a plausible intervening mechanism between family structure and children’s BMI.

Building on the evidence linking family structure, socioeconomic disadvantage, and childhood obesity, the present study aimed to examine whether SES serves as a mechanism underlying the association between family structure and children’s BMI.

Specifically, this study investigated whether children living in single-parent families experience less favorable socioeconomic circumstances and whether these socioeconomic differences are associated with variations in BMI z-scores. Based on previous research, three hypotheses were formulated. First, it was hypothesized that children living in single-parent families would have lower SES than children living in two-parent families (H1). Second, higher SES was expected to be associated with lower BMI z-scores among children (H2). Third, SES was hypothesized to mediate the association between family structure and children’s BMI z-scores, such that children living in single-parent families would have lower SES, which in turn would be associated with higher BMI z-scores (H3). By examining these relationships in a nationally representative sample, the study seeks to provide a better understanding of the socioeconomic pathways linking family structure to childhood weight outcomes.

## 2. Materials and Methods

### 2.1. Study Design and Participants

The present study was based on data obtained from the Croatian Childhood Obesity Surveillance Initiative (CroCOSI), which represents Croatia’s contribution to the World Health Organization (WHO) Childhood Obesity Surveillance Initiative [42,43]. CroCOSI is a population-based surveillance program designed to monitor childhood overweight and obesity and their determinants using harmonized procedures implemented across participating European countries.

A cross-sectional design was employed. Participants were selected through a stratified cluster sampling procedure intended to ensure national representativeness of primary school children in Croatia. The sampling frame consisted of second- and third-grade pupils attending public primary schools throughout the country. Schools and classes were selected according to the sampling methodology prescribed by the WHO COSI protocol [44].

**Figure 1:**
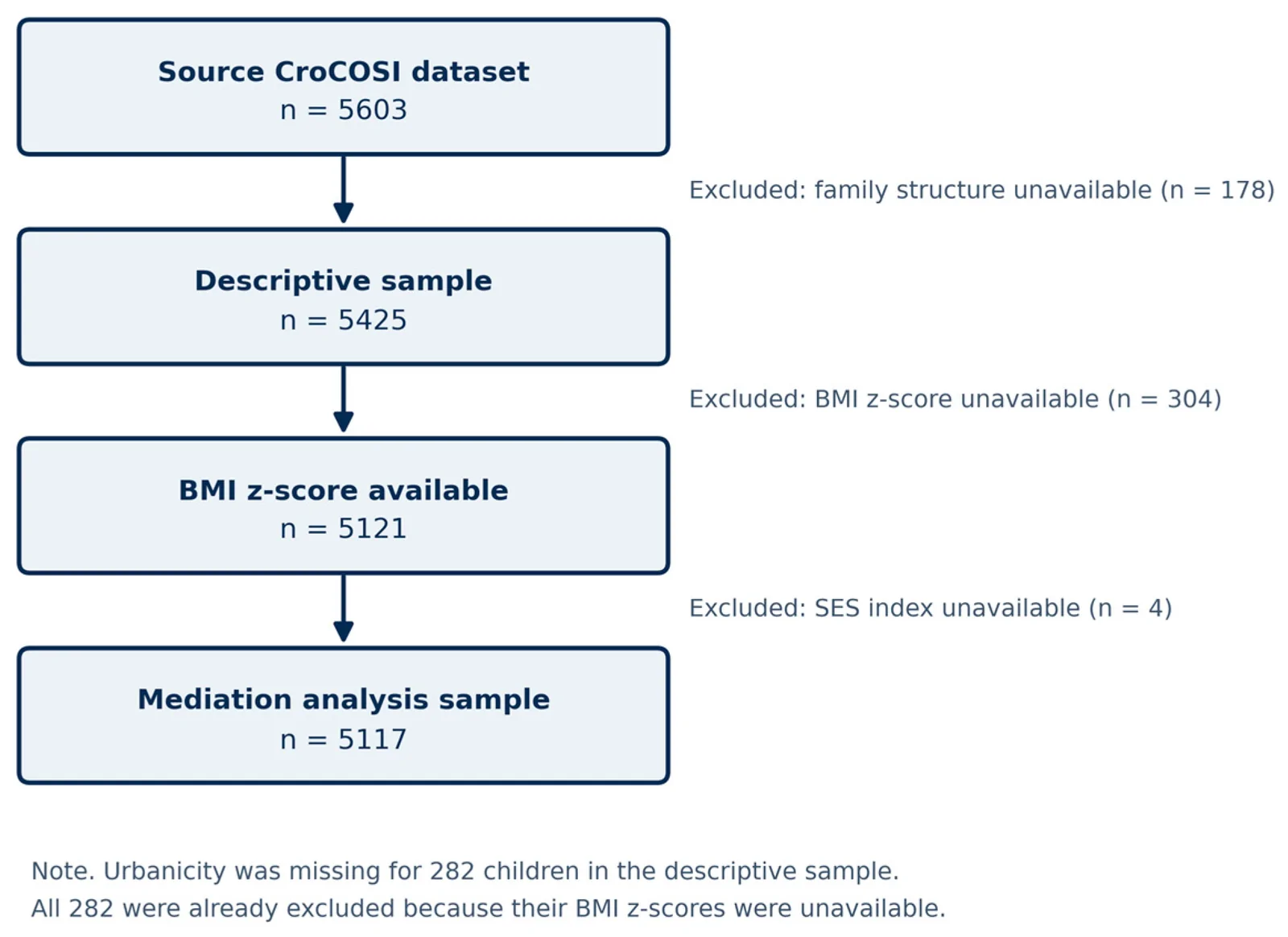
Flowchart of participant selection for the descriptive and mediation analyses, *n* = number of children; BMI = body mass index; SES = socioeconomic status.

The source CroCOSI dataset comprised 5603 children. Family structure information was unavailable for 178 children, leaving 5425 children for the descriptive analyses. Of these, 304 had no BMI z-score. Among the remaining 5121 children, four had no SES index. The complete-case sample for the mediation analysis therefore comprised 5117 children. Missing urbanicity data for 282 children did not lead to additional exclusions because all 282 were already excluded owing to missing BMI z-scores. The participant selection process is shown in Figure 1.

Participation was voluntary and required written informed consent from parents or legal guardians. All study procedures were conducted in accordance with national ethical requirements.

### 2.2. Data Collection Procedure

Data collection was carried out during the 2021/2022 school year following the standardized operational procedures of the WHO COSI programme [44,45]. Information was gathered from multiple sources, including direct anthropometric assessments of children and questionnaires completed by parents or caregivers.

Prior to data collection, field staff received training in standardized measurement procedures. Children’s body height and weight were measured at school using calibrated instruments and according to established WHO protocols. These measurements were subsequently used to calculate body mass index and age-standardized BMI z-scores.

Information regarding household composition, parental education, household financial circumstances, and other sociodemographic characteristics was obtained from questionnaires completed by parents or primary caregivers. The questionnaires were distributed through participating schools together with information about the study and consent forms. Completed questionnaires were returned to schools and collected by trained research personnel.

To ensure comparability with data collected in other participating countries, all study instruments and procedures followed the methodological guidelines established by the WHO Regional Office for Europe [44].

### 2.3. Measures

Family Structure

Family structure was assessed using information provided in the Family Record Form. Children were classified into two groups: those living in two-parent families and those living in single-parent families. This variable served as the independent variable in the mediation analysis.

Body Mass Index z-score

Children’s body mass index (BMI) was calculated as weight in kilograms divided by height in meters squared (kg/m^2^). BMI values were transformed into age- and sex-standardized BMI z-scores using WHO growth reference data for children aged 5–19 years. BMI z-score served as the outcome variable in all analyses.

Socioeconomic Status

SES was operationalized as a composite index based on parental educational attainment and household financial circumstances. Parental educational attainment was represented by the highest educational level reported for either parent. Household financial circumstances were assessed using a four-category item evaluating the household’s ability to cover monthly expenses. Responses were recoded so that higher values indicated more favorable financial conditions. Both indicators were standardized prior to index construction. The SES index was calculated as the mean of the standardized parental education and household financial circumstances scores, with higher values indicating more favorable socioeconomic conditions.

Covariates

Four covariates were included in the mediation analysis: child age (in years), gender, region of residence, and urbanicity. Region was classified into four geographical regions of Croatia (City of Zagreb, Adriatic, Northern, and Pannonian regions). Urbanicity was categorized as urban, settlement, or rural area.

### 2.4. Statistical Analysis

All analyses were conducted using IBM SPSS Statistics 27 and the PROCESS macro (Version 5.0) [46]. Statistical significance was set at *p* < 0.05. Post-stratification weights, created by the European Office of the World Health Organization to account for the sampling design, oversampling, and nonresponse, were applied in all analyses to allow inference from the sample to the population. Weighted and unweighted analyses yielded substantively similar results; therefore, only weighted results are reported.

Descriptive statistics were calculated for all study variables. Differences between children from single-parent and two-parent families were examined using independent-samples *t*-tests for continuous variables and Pearson’s χ^2^ tests for categorical variables. Bivariate associations among family structure, SES, and BMI z-score were examined using Pearson correlation coefficients.

The hypothesized mediation model was tested using PROCESS Model 4. Family structure was specified as the independent variable, SES as the mediator, and BMI z-score as the outcome variable. Age, gender, region, and urbanicity were included as covariates. Indirect effects were estimated using 5000 bootstrap samples, and statistical significance was determined using bias-corrected 95% confidence intervals. An indirect effect was considered statistically significant when the confidence interval did not include zero. To assess potential differences associated with exclusion from the mediation analysis, we compared included and excluded children on available BMI z-scores using Welch’s *t*-test and on family structure using Pearson’s chi-square test.

## 3. Results

Before examining the study variables, we compared children included in the mediation analysis with those excluded from the source dataset. The groups did not differ significantly in their available BMI z-scores (included: *n* = 5117, *M* = 0.591, *SD* = 1.315; excluded: *n* = 175, *M* = 0.677, *SD* = 1.338; Welch’s *p* = 0.404) or in the proportion living in single-parent families (included: 499/5117, 9.75%; excluded: 32/308, 10.39%; χ^2^ *p* = 0.714).

Descriptive statistics and group comparisons by family structure are presented in Table 1. Of the total sample, 4894 children (90.2%) lived in two-parent families, and 531 children (9.8%) lived in single-parent families.

No significant difference in BMI z-scores was observed between children from two-parent and single-parent families (*t* = −1.36, *p* = 0.173). Significant differences were observed across socioeconomic indicators. Compared with children from two-parent families, those living in single-parent family had lower SES index scores (*t* = 9.85, *p* < 0.001), lower parental educational attainment (*t* = 6.18, *p* < 0.001), and less favorable household financial circumstances (*t* = 9.17, *p* < 0.001). The latter two variables constitute the SES index used in the mediation analysis.

Age, gender, urbanicity, and region were included as covariates in the mediation model. No significant differences between family structure groups were found for gender (χ^2^ = 0.08, *p* = 0.776), urbanicity (χ^2^ = 2.70, *p* = 0.259), or region (χ^2^ = 4.84, *p* = 0.184). However, children living in single-parent families were slightly older than those living in two-parent families (*t* = −3.23, *p* = 0.001).

Bivariate correlations among family structure, SES, and BMI z-score are presented in Table 2. Family structure was negatively associated with SES (*r* = −0.136, *p* < 0.001), while SES was negatively associated with children’s BMI z-score (*r* = −0.084, *p* < 0.001). The correlation between family structure and BMI z-score was not statistically significant (*r* = 0.019, *p* > 0.05). Overall, the pattern of correlations was consistent with the hypothesized mediation model and provided preliminary support for the proposed indirect association between family structure and BMI z-score through SES.

A mediation analysis was conducted to examine whether SES mediates the association between family structure and children’s BMI z-scores. The results are presented in Table 3 and Table 4. As shown in Table 4, single-parent family structure was significantly associated with lower SES (B = −0.375, SE = 0.036, *t* = −10.50, *p* < 0.001). SES was significantly associated with children’s BMI z-scores, with higher SES being related to lower BMI z-scores (B = −0.114, SE = 0.024, *t* = −4.75, *p* < 0.001). The direct effect of family structure on BMI z-score was not statistically significant after accounting for SES and the covariates (B = 0.051, SE = 0.062, *t* = 0.82, *p* = 0.413).

Bootstrap analyses based on 5000 resamples indicated a statistically significant indirect effect of family structure on BMI z-score through SES (B = 0.043, SE = 0.010, 95% CI [0.024, 0.065]). Because the confidence interval did not include zero, the indirect effect was considered statistically significant.

Regarding the covariates, urbanicity (B = 0.053, SE = 0.027, *t* = 1.98, *p* = 0.048) and gender (B = −0.280, SE = 0.036, *t* = −7.68, *p* < 0.001) were significantly associated with BMI z-score, whereas age (B = −0.022, SE = 0.030, *t* = −0.72, *p* = 0.469) and region (B = 0.033, SE = 0.020, *t* = 1.66, *p* = 0.097) were not. In the SES model, age (B = −0.063, SE = 0.017, *t* = −3.63, *p* < 0.001), region (B = −0.086, SE = 0.012, *t* = −7.44, *p* < 0.001), and urbanicity (B = −0.170, SE = 0.015, *t* = −11.11, *p* < 0.001) were significant predictors of SES, whereas gender was not significantly associated with SES (B = −0.005, SE = 0.021, *t* = −0.24, *p* = 0.809). Taken together, these findings support the hypothesized indirect association between single-parent family structure and children’s BMI z-scores through SES.

## 4. Discussion

The present study examined whether SES mediates the association between single-parent family structure and children’s BMI z-scores in a nationally representative sample of Croatian school-aged children. Three main findings emerged. First, children living in single-parent families exhibited lower SES than those living in two-parent families. Second, higher SES was associated with lower BMI z-scores. Third, SES significantly mediated the association between family structure and BMI z-score. Specifically, children living in single-parent families tended to experience less favorable socioeconomic circumstances, which in turn were associated with higher BMI z-scores. Notably, the direct association between family structure and BMI z-score was not statistically significant after accounting for SES, suggesting that socioeconomic disadvantage may represent an important mechanism linking single-parent family structure to childhood weight outcomes.

These findings should be interpreted in the context of the rate of childhood obesity in Croatia, which is among the highest in Europe. In the WHO Childhood Obesity Surveillance Initiative (CroCOSI 2018/2019), 35% of children aged 8–9 years had overweight or obesity [43,47,48,49]. National data from 2003 to 2022 show that overweight, including obesity, affected 27.9% of primary school children in 2021/2022. They also show marked regional differences: prevalence was lowest in the City of Zagreb and highest in the Adriatic region [43]. Notably, the authors of that analysis could not examine socioeconomic inequalities because individual socioeconomic data were not available [43]. By using individual-level SES measures in a nationally representative sample, the present study helps to fill this gap.

Consistent with the first hypothesis, children living in single-parent families had significantly lower SES than children living in two-parent families. This finding is in line with a substantial body of international research showing that single-parent households are more likely to experience socioeconomic disadvantage, including lower educational attainment, reduced household income, and an elevated risk of poverty [50,51]. The disadvantage is particularly pronounced in Croatia. In 2025, the at-risk-of-poverty rate was 29.7% for single-parent households with dependent children, compared with 12.2% for two-adult households with dependent children [52]. Single-parent families in Croatia are therefore more than twice as likely to live in poverty. Nieuwenhuis and Maldonado (2015) describe the situation of these families as a “triple bind” of inadequate resources, limited employment opportunities and insufficient policy support [51]. The concentration of these multiple disadvantages helps explain why SES emerges as such a powerful mediator in the relationship between family structure and child health outcomes, as observed in our study.

The second hypothesis was also supported. Higher SES was associated with lower BMI z-scores among children. This finding is consistent with evidence from high-income countries indicating that children from socioeconomically advantaged families generally experience a lower risk of overweight and obesity [53,54,55]. Several mechanisms may explain this relationship. Families with greater socioeconomic resources are often better positioned to provide healthier diets, support participation in organized physical activities, access preventive healthcare services, and create environments that promote healthy lifestyles [55]. Conversely, socioeconomic disadvantage may increase exposure to obesogenic environments and limit opportunities for healthy behavioral choices. The present results therefore reinforce the importance of socioeconomic inequalities as a determinant of childhood weight status.

The third hypothesis received support. SES significantly mediated the association between family structure and children’s BMI z-scores. Children living in single-parent families tended to have lower SES, and this socioeconomic disadvantage was associated with higher BMI z-scores. Importantly, the direct association between family structure and BMI z-score was not statistically significant once SES was included in the model. This pattern of findings suggests that family structure itself may not be the primary factor associated with children’s weight outcomes. Rather, the socioeconomic conditions that frequently accompany single-parent family arrangements appear to be a more relevant explanatory mechanism. However, a non-significant direct effect does not rule out other pathways, such as parenting practices, family routines and stress, which may operate alongside SES.

Only a few studies have formally examined the mechanisms linking family structure to children’s weight, and their findings are mixed. In a comparative study of 11-year-old children in Australia, the United Kingdom and the United States, Slighting et al. found that most of the association between family structure and child overweight or obesity was explained by differences in families’ access to resources, including income, parental education and employment [56]. This is consistent with our finding that SES accounted for the association. The authors also observed that these associations differed between countries and were weakest in Australia, which they attributed to its stronger social safety net [56], supporting the view that the mediating role of SES depends on the national policy context. In contrast, Goisis et al., using longitudinal data from the UK Millennium Cohort Study, found that parental separation was followed by a gradual increase in children’s BMI that was not attenuated by family income, parental depression or health behaviors [57]. Similarly, Tosi reported a persistent increase in BMI after parental separation among children in the United States, with larger effects among children of less-educated mothers [58].

Several differences may explain these divergent findings. First, the longitudinal studies examined parental separation as an event and followed changes in weight over time, whereas we compared children living in single-parent and two-parent families at one point in time. Our results may therefore partly reflect pre-existing socioeconomic differences between these families rather than the consequences of separation itself. Second, Tosi’s finding that maternal education moderated the effect of separation [58] suggests that SES may act not only as a mediator, but also as a buffer against the adverse effects of family disruption. Taken together, the evidence indicates that socioeconomic resources are a central, though probably not the only, pathway linking family structure to children’s weight.

The present study extends this literature in two ways. Whereas most previous studies have reported direct associations between single-parent family structure and childhood obesity [59,60], we formally tested SES as a mediator. These findings suggest that policies designed to reduce socioeconomic inequalities and improve the living conditions of single-parent families may be a promising strategy for addressing disparities in childhood obesity.

The Croatian policy context may also shape these associations. Since January 2023, all primary school pupils have been entitled to free school meals; previously, about 30% of children received them [61]. Although this universal measure may have narrowed dietary differences between children, a clear SES gradient in BMI remained, suggesting that other socioeconomic pathways are involved. The mediating role of SES may also differ in countries with stronger income support for single-parent families, as suggested by the cross-national comparison of Slighting et al. [56].

Several limitations should be considered when interpreting the present findings. First, the study employed a cross-sectional design, which precludes causal inference. Although the proposed mediation model was theoretically grounded and consistent with previous research, the temporal ordering of family structure, SES, and BMI cannot be established with certainty. Future longitudinal studies are needed to examine whether socioeconomic disadvantage prospectively mediates the association between family structure and children’s weight outcomes.

Second, SES was operationalized using a composite index based on parental educational attainment and perceived household financial circumstances. While these indicators capture important dimensions of socioeconomic disadvantage, SES is a multidimensional construct that may also encompass additional factors, such as household income, occupational status, and material deprivation. Future research should consider a broader range of socioeconomic indicators to better understand the mechanisms linking family structure and childhood obesity.

Third, information on parental education, household financial circumstances, and family structure was obtained through parent-reported questionnaires. Although such measures are commonly used in population-based studies, they may be subject to reporting bias and measurement error.

Fourth, the study focused exclusively on SES as a potential explanatory mechanism. Other pathways linking family structure to children’s weight outcomes, including dietary habits, physical activity, sedentary behavior, sleep patterns, and parenting practices, were not examined. Future studies should investigate these factors simultaneously to provide a more comprehensive understanding of the processes underlying socioeconomic inequalities in childhood obesity.

Finally, children were nested within schools, and, given the marked regional differences in obesity prevalence in Croatia [43], future studies should use multilevel models to account for school- and region-level factors.

The present findings have important implications for public health policy and childhood obesity prevention efforts. The results suggest that socioeconomic disadvantages, rather than family structure itself, may represent the primary mechanism linking single-parent families to children’s BMI. Consequently, interventions aimed at reducing childhood obesity should pay particular attention to the socioeconomic challenges faced by single-parent families. Measures that improve financial security for single parents, such as targeted income support and affordable childcare, may help reduce inequalities in children’s weight. Universal measures such as free school meals and community-based programs may play an important role in supporting children from socioeconomically disadvantaged households by promoting healthy nutrition, physical activity, and other health-related behaviors. More broadly, the findings highlight the importance of addressing social determinants of health in national strategies as part of comprehensive strategies to prevent childhood obesity.

## 5. Conclusions

The study examined the role of SES in the association between single-parent family structure and children’s BMI z-scores using data from a nationally representative sample of Croatian school-aged children. The findings showed that children living in single-parent families experienced less favorable socioeconomic conditions and that lower SES was associated with higher BMI z-scores. Moreover, SES significantly mediated the relationship between family structure and children’s BMI, whereas the direct association between family structure and BMI was not statistically significant. These results suggest that socioeconomic disadvantage represents an important pathway linking single-parent family structure to childhood weight outcomes. Addressing socioeconomic inequalities may therefore be a key component of efforts aimed at reducing disparities in childhood obesity and promoting healthier developmental trajectories among children.

## Figures and Tables

**Table 1:** Sample characteristics according to family structure.

Variable	Total (*n* = 5425)	Two-Parent Family (*n* = 4894)	Single-Parent Family (*n* = 531)		
**Continuous variables**	***M (SD)***	***M (SD)***	***M (SD)***	***t***	***p***
BMI z-score	0.59 (1.32)	0.58 (1.31)	0.67 (1.35)	−1.36	0.173
Child age (years)	9.04 (0.61)	9.03 (0.61)	9.12 (0.62)	−3.23	0.001
SES index	0.00 (0.79)	0.04 (0.78)	−0.32 (0.82)	9.85	<0.001
Highest parental education	3.89 (0.97)	3.92 (0.97)	3.65 (0.94)	6.18	<0.001
Household financial situation	3.35 (0.76)	3.38 (0.74)	3.06 (0.86)	9.17	<0.001
**Categorical variables**	***n (%)***	***n (%)***	***n (%)***	***χ*** **^2^**	*p*
**Gender**				0.08	0.776
Boys	2788 (51.4)	2512 (51.3)	276 (52.0)		
Girls	2637 (48.6)	2382 (48.7)	255 (48.0)		
**Urbanization level**				2.7	0.259
Urban	1944 (37.8)	1738 (37.4)	206 (41.0)		
Settlement	1665 (32.4)	1507 (32.5)	158 (31.5)		
Rural	1534 (29.8)	1396 (30.1)	138 (27.5)		
**Region**				4.84	0.184
City of Zagreb	1185 (21.8)	1062 (21.7)	123 (23.2)		
Adriatic region	1854 (34.2)	1657 (33.9)	197 (37.1)		
Northern region	1029 (19.0)	943 (19.3)	86 (16.2)		
Pannonian region	1357 (25.0)	1232 (25.2)	125 (23.5)		

*Note.* The table describes 5425 children with available family structure data. The number of available observations varies by variable: BMI z-score, *n* = 5121; age, gender, and region, *n* = 5425; SES index, *n* = 5421; highest parental education, *n* = 5409; household financial situation, *n* = 5363; and urbanicity, *n* = 5143. Percentages for categorical variables are based on the available observations in the respective column. SES = socioeconomic status index based on parental education and household financial situation. Continuous variables are presented as means and standard deviations. Group differences were examined using independent-samples *t*-tests and Pearson’s χ^2^ tests. M = mean; SD = standard deviation; t = independent-samples *t*-test statistic; *p* = *p*-value; *n* = number of children; χ^2^ = Pearson’s chi-square test statistic. BMI = body mass index.

**Table 2:** Pearson correlations among family structure, SES, and children’s BMI z-score.

	1	2	3
1. BMI z-score	—		
2. Single-parent family structure	0.019	—	
3. SES	−0.084 *	−0.136 *	—

*Note*. *N* = 5117 children with complete data for the mediation model. SES = SES index based on parental education and household financial situation. Higher values indicate more favorable socioeconomic conditions, * *p* < 0.001. BMI = body mass index.

**Table 3:** Mediation analysis of SES in the association between family structure and BMI z-score.

Effect	B	SE	LLCI	ULCI
Indirect effect via SES	0.043	0.01	0.024	0.065
Direct effect	0.051	0.062	−0.071	0.173

*Note*. *N* = 5117 children with complete data for the mediation model. SES = socioeconomic status index based on parental education and household financial situation. Indirect effects were estimated using PROCESS Model 4 with 5000 bootstrap samples. Age, region, urbanicity, and gender were included as covariates. B = unstandardized regression coefficient; SE = standard error; LLCI = lower limit confidence interval; ULCI = upper limit confidence interval.

**Table 4:** Regression coefficients for the mediation model.

	B	SE	t	*p*
Outcome: SES				
Single-parent family structure	−0.375	0.036	−10.5	<0.001
Age	−0.063	0.017	−3.63	<0.001
Region	−0.086	0.012	−7.44	<0.001
Urbanicity	−0.17	0.015	−11.11	<0.001
Gender	−0.005	0.021	−0.24	0.809
Outcome: BMI z-score				
Single-parent family structure	0.051	0.062	0.82	0.413
SES	−0.114	0.024	−4.75	<0.001
Age	−0.022	0.03	−0.72	0.469
Region	0.033	0.02	1.66	0.097
Urbanicity	0.053	0.027	1.98	0.048
Gender	−0.28	0.036	−7.68	<0.001

*Note*. B = unstandardized regression coefficient; SE = standard error; t = t-statistic; *p* = *p*-value; SES = socioeconomic status index based on parental education and household financial situation; BMI = body mass index.

## Data Availability

The datasets generated and/or analysed during the current study are available in the [CroCOSI survey] repository https://www.hzjz.hr/wp-content/uploads/2021/03/CroCOSI-publikacija-2021-ENGLESKI-WEB-pages.pdf (accessed on 28 September 2026) (ii) the datasets generated and/or analysed during the current study are available from the corresponding author upon reasonable request.
